# Production of Live Offspring from Vitrified-Warmed Oocytes Collected at Metaphase I Stage

**DOI:** 10.1371/journal.pone.0157785

**Published:** 2016-06-22

**Authors:** Ching-Chien Chang, Wei-Fang Chang, Jie Xu, An-Sheng Cheng, Chia-Chun Chang, Zsolt Peter Nagy, Cho-Chen Yang, Shih-Torng Ding, Li-Ying Sung

**Affiliations:** 1 Reproductive Biology Associates, Atlanta, Georgia, United States of America; 2 Institute of Biotechnology, National Taiwan University, Taipei, Taiwan; 3 Center for Advanced Models for Translational Sciences and Therapeutics, University of Michigan Medical Center, Ann Arbor, Michigan, United States of America; 4 Department of Medicinal Plant Development, Yupintang Traditional Chinese Medicine Foundation, Kaohsiung City, Taiwan; 5 Department of Animal Science, National Chiayi University, Chiayi City, Taiwan; 6 Department of Animal Science and Technology, National Taiwan University, Taipei, Taiwan; 7 Agricultural Biotechnology Research Center, Academia Sinica, Taipei, Taiwan; 8 Animal Resource Center, National Taiwan University, Taipei, Taiwan; University of Connecticut, UNITED STATES

## Abstract

Vitrification of matured oocytes is widely adopted in human clinics and animal research laboratories. Cryopreservation of immature oocytes, particularly those at metaphase I (MI), remains a challenge. In the present work, mouse MI oocytes denuded of cumulus cells were vitrified and warmed (V/W) either prior to (V/W-BEFORE-IVM, n = 562) or after (V/W-AFTER-IVM, n = 664) *in vitro* maturation (IVM). Derivative metaphase II (MII) oocytes were then used for intracytoplasmic sperm injection (ICSI). In the control groups, *in vivo* matured MII oocytes were used freshly (FRESH-MII, n = 517) or after V/W (MII-V/W, n = 617). *In vitro* and *in vivo* developmental competencies were compared among groups. Satisfactory blastocyst rates were achieved in V/W-BEFORE-IVM (27.5%) and V/W-AFTER-IVM (32.4%) groups, albeit as expected still lower than those from fresh-MII (56.1%) or MII-V/W (45.6%) oocytes. Similarly, the term development rates from V/W-BEFORE-IVM and V/W-AFTER-IVM were 12.4% and 16.7% respectively, acceptable but lower than those of the fresh-MII (41.2%) and MII-V/W (23.3%) groups. These data demonstrate that oocytes collected at MI stage are amenable to V/W, which can be performed before or after IVM with acceptable development rates including production of healthy pups. These findings provide useful knowledge to researchers and clinical practitioners for preservation and use of the otherwise discarded MI oocytes.

## Introduction

Conventional slow freezing and vitrification are common methods to preserve mammalian gametes and embryos. Vitrification, defined as the solidification of a solution at low temperature by extreme elevation in viscosity during cooling without ice crystallization, has been increasingly used in the past two decades. The glass state formed during vitrification has the same ionic and molecular distribution as the liquid phase, thus avoiding both chemical and mechanical damage to gametes and embryos. Importantly, vitrification has been proven effective on cryopreserving matured female gametes, often leading to minor compromise of the fertility and developmental competency; consequently it is widely adopted in human IVF clinics [1–9] and in animal research laboratories [10–12].

While vitrification of MII oocytes is reliable, it is noted that a working protocol to vitrify immature oocytes remains to be developed. Notably, 15–30% oocytes retrieved in a routine human IVF cycle are immature and discarded [13, 14] due to lack of such protocols. One important parameter is the timing of vitrification with regard to IVM: (1) V/W is conducted prior to IVM; or (2) IVM is conducted before V/W. It is argued that the maturation status may render differential cryotolerance to the oocytes [15]. Most previous studies used germinal vesicle (GV) stage oocytes to do the comparisons [16–21]. The effects of vitrification timing on MI oocytes have not been evaluated, and there are no reports on production of live offspring using oocytes V/W at MI stage.

**Fig 1:**
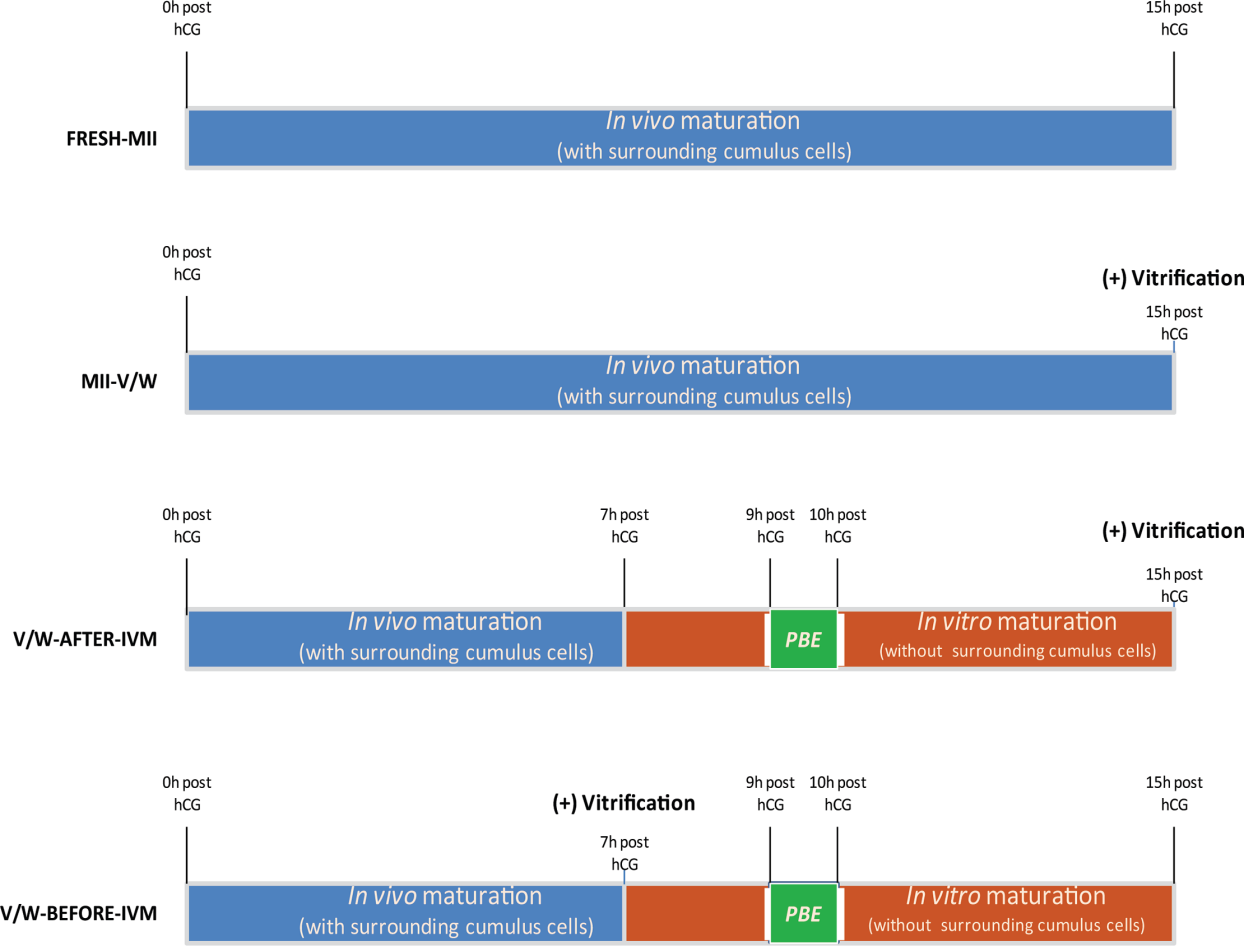
Schematic illustration of treatments in FRESH-MII (Control 1), VW-MII (Control 2), V/W-AFTER-IVM, and V/W-BEFORE-IVM groups. The duration of oocyte maturation was 15 hours in all groups, which used hCG trigger as a set point. In FRESH-MII group, the freshly collected IVO MII oocytes were injected with a spermatozoa without any vitrification. In VW-MII Group, IVO MII oocytes were harvested 15 h after the hCG trigger from the oviducts, and those IVO MII oocytes were vitrified for later ICSI. In V/W-AFTER-IVM Group, cumulus cells were removed from MI oocytes, which collected 6–7 h after the hCG trigger from ovaries. After another 8 h of *in vitro* maturation, the oocytes with a PB extruded (IVM MI-II oocytes) were vitrified for later ICSI. In V/W-BEFORE-IVM Group, cumulus cells removed MI oocytes, which collected 6–7 h after the hCG trigger from ovaries, were vitrified for storage. After oocyte warming, the MI oocytes were *in vitro* matured for another 8 h, and the oocytes with a PB extruded (IVM MI-II) were regarded as mature oocytes for ICSI.

In the present work, we conducted V/W on MI oocytes at two time points: (1) before IVM; and (2) after IVM (Fig 1). Derivative MII oocytes from both groups, named V/W-BEFORE-IVM and V/W-AFTER-IVM respectively, were subjected for fertilization. Blastocyst rates and term rates after embryo transfer (ET) were used to evaluate the *in vitro* and in vivo developmental competencies of these oocytes, in comparison to the two control groups, fresh MII (FRESH-MII) or vitrified-warmed MII (MII-V/W) oocytes.

## Material and Methods

### Animals

All animal maintenance, care and use procedures were reviewed and approved by the Institutional Animal Care and Use Committee (NTU-103-EL-47) of National Taiwan University, Taiwan. The 8–12 weeks-old B6D2F1 hybrid mice used as sperm and oocyte donors for ICSI were from C57BL/6 (B6) females breed with DBA/2 males, the female CD1 mice were used as recipient mothers.

### Groups of oocytes

A schematic illustration of the oocyte groups is shown in Fig 1. In **FRESH-MII** Group, in vivo matured (IVO) MII oocytes were harvested from the oviducts 15 h after the hCG trigger, and were injected with a spermatozoa without any vitrification. In **MII-V/W** Group, IVO MII oocytes were harvested from the oviducts 15 h after the hCG trigger, and were immediately vitrified, stored in liquid nitrogen until they were warmed (or interchangeably referred to as “thawed” in this manuscript) for ICSI at a later time. In **V/W-AFTER-IVM** Group, cumulus cells were removed from MI oocytes, which were collected 6–7 h post hCG trigger. After another 8 h of *in vitro* maturation in KSOM+AA medium, the oocytes with an extruded PB (presumably MII) were selected, vitrified and stored, before they were warmed at a later date for ICSI. In **V/W-BEFORE-IVM** Group, MI oocytes were collected 6–7 h after the hCG trigger, denuded of cumulus cells, vitrified and stored before they were warmed at a later date, *in vitro* matured for another 8 h in KSOM+AA medium; oocytes with an extruded PB (presumably MII) were selected for ICSI. In V/W-BEFORE-IVM and V/W-AFTER-IVM groups, cumulus cells were removed to simulate clinical applications in human ICSI practices in which (undetermined) immature oocytes are denuded of cumulus cells upon retrieval.

### Collection of metaphase II and metaphase I oocytes

#### *In vivo* matured metaphase II (IVO MII) oocytes

**Fig 2:**
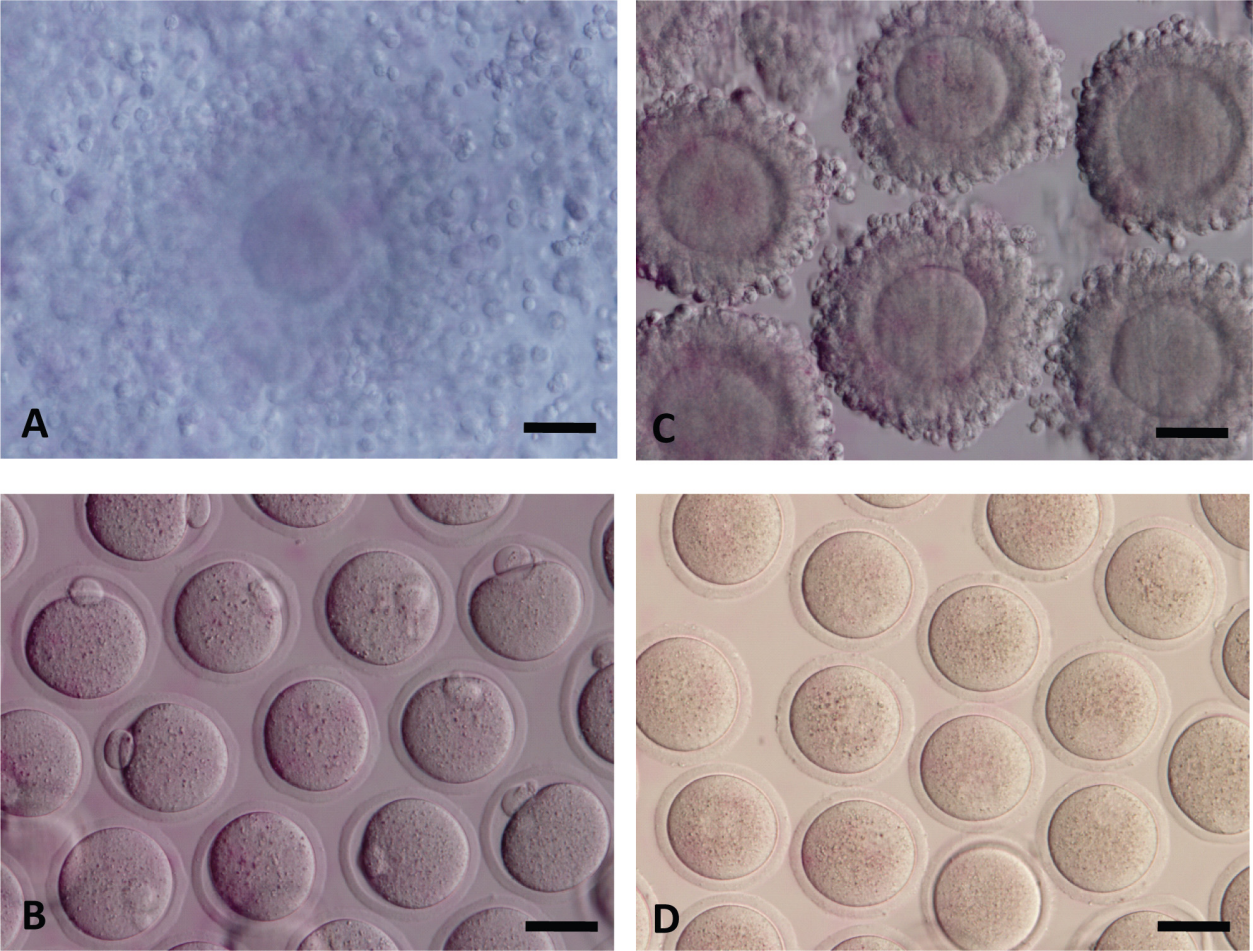
Collection of metaphase oocytes at different time points. (A) MII COCs were harvested 15 hours after hCG trigger from oviducts. (B) The cumulus cells were removed from MII COCs. (C) MI COCs were harvested 6–7 hours after hCG trigger from ovaries. (D) The cumulus cells were removed from MI COCs. Scale bar = 50 μm.

B6D2F1 female mice were subjected to the following hormone priming protocol: superovulation was induced with 5 IU of equine chorionic gonadotrophin (eCG) followed 48 h later with 5 IU of human chorionic gonadotrophin (hCG). Oocytes at the MII stage were harvested 15 h after the hCG trigger from oviducts and denuded of cumulus cells by 2-min exposure to 0.1 mg/mL of hyaluronidase at 37°C with gentle pipetting (Fig 2A). Oocytes with an extruded PB (Fig 2B) were used for ICSI either freshly (FRESH-MII), or after vitrify-warming (VW-MII).

#### V/W-AFTER-IVM oocytes

B6D2F1 female mice were subjected to the following hormone priming protocol: superovulation was induced with 5 IU of equine chorionic gonadotrophin (eCG) followed 48 h later with 5 IU of human chorionic gonadotrophin (hCG). Oocytes at the MI stage (Fig 2C) were collected 6–7 h after the hCG trigger from ovaries and removed cumulus cells by 2 min exposure to 0.1 mg/mL of hyaluronidase at 37°C with pipetting (Fig 2D). After another 8 h of culture *in vitro*, oocytes with an extruded PB were vitrified (V/W-AFTER-IVM) and stored before they were warmed on a later date for ICSI.

#### V/W-BEFORE-IVM oocytes

B6D2F1 female mice were subjected to the following hormone priming protocol: superovulation was induced with 5 IU of equine chorionic gonadotrophin (eCG) followed 48 h later with 5 IU of human chorionic gonadotrophin (hCG). Oocytes at the MI stage were collected 6–7 h after the hCG trigger from ovaries and removed cumulus cells by 2 min exposure to 0.1 mg/mL of hyaluronidase at 37°C with pipetting (Fig 2D). The denuded MI oocytes were vitrified for storage. After warming, the MI oocytes were *in vitro* matured for another 8 h, and oocytes with an extruded PB (V/W-BEFORE-IVM).

### Vitrification of oocytes

The basal medium used for oocyte cryopreservation was HEPES-buffered human tubal fluid (HTF) supplemented with 20% (v/v) fetal bovine serum (FBS; SH0070.03; Hyclone, Logan, UT, USA). The denuded oocytes were vitrified by the minimum volume cooling method as described previously [1, 12]. Briefly, the oocytes were equilibrated in equilibration medium [basal medium with 7.5% (v/v) ethylene glycol and 7.5% (v/v) dimethylsulphoxide (DMSO)] at room temperature for 5 min. The oocytes were then transferred into the vitrification medium [basal medium with 15% (v/v) ethylene glycol, 15% (v/v) DMSO, and 0.5 mol/L sucrose] at room temperature (RT) for 45–60 sec. The cryoprotectant-treated oocytes were placed onto a fine polypropylene strip (Cryotop**®**, Kitazato BioPharma Co., Fuji, Shizuoka, Japan). The polypropylene strip carrying the oocytes was then submerged immediately into liquid nitrogen and was ready for storage.

### Warming procedures

The polypropylene strip with vitrified oocytes was immersed directly into 3.5 mL of thawing solution [HEPES buffered HTF with 20% (v/v) FBS and 1.0 mol/L sucrose] at 37°C for 1 min. Oocytes were then picked up and transferred into 1.0mL of the dilution solution [HEPES-buffered HTF with 20% (v/v) FBS and 0.5 mol/L sucrose] for 3 min at RT. The oocytes were subsequently washed in 1.0mL washing solution [HEPES-buffered HTF with 20% (v/v) FBS] for 10 min at RT. Finally, the oocytes were incubated in KSOM**+**AA medium (Specialty Media, Phillipsburg, NJ, USA) allow recover for at least 1h before sperm injection.

### Intracytoplasmic sperm injection

ICSI was performed as described by Kishigami *et al* [22]. Briefly, mature spermatozoa were obtained from the epididymides of 8–12 weeks old B6D2F1 male. A cauda epididymis was removed and placed into a 1.5-ml polypropylene centrifuge tube contents 500 ml of KSOM+AA medium, then incubated for 25~30 min at 37°C to allow the spermatozoa to swam into the medium. A small drop (about 2μl) of sperm suspension from upper part was mixed with about 10 μl of HEPES-CZB medium containing 12% (w/v) polyvinylpyrrolidone (PVP; 360 kDa) in a micromanipulation chamber. To increase the survival rate after sperm injection, the oocytes were incubated in calcium-free CZB medium containing 5 mM strontium for 20 min before ICSI [22]. Following activation of oocytes, the head of a spermatozoon was injected into the oocyte by using a pipette with an inner diameter of 6–8 μm assisted by piezo-drill pulses. Injected oocytes were left for 10–15 min on the stage of the microscope at room temperature, and then further cultured in KSOM+AA medium for 4 days at 37°C in 5% CO_2_ humidified air. Cleavage and blastocyst rates were recorded 1 day and 4 days post culture.

### Embryo transfer

The embryos that developed to the blastocyst stage were transferred into the uteri of day 2.5 pseudopregnant CD1 females mated with vasectomized males. The live pups were obtained through a natural birth from recipient mothers around 17 days later following transfer and raised by recipient mothers.

### Statistical analyses

Percentages were transformed using arcsin transformation. Percentage transformed data and other numbers were analyzed by ANOVA and means compared by Fisher’s LSD using Graphpad Prism (v6.02, Graphpad Software, Inc., La Jolla, CA). Significant differences were defined as P<0.05 (*).

## Results

### Oocyte survival and *in vitro* embryo development after warming

**Fig 3:**
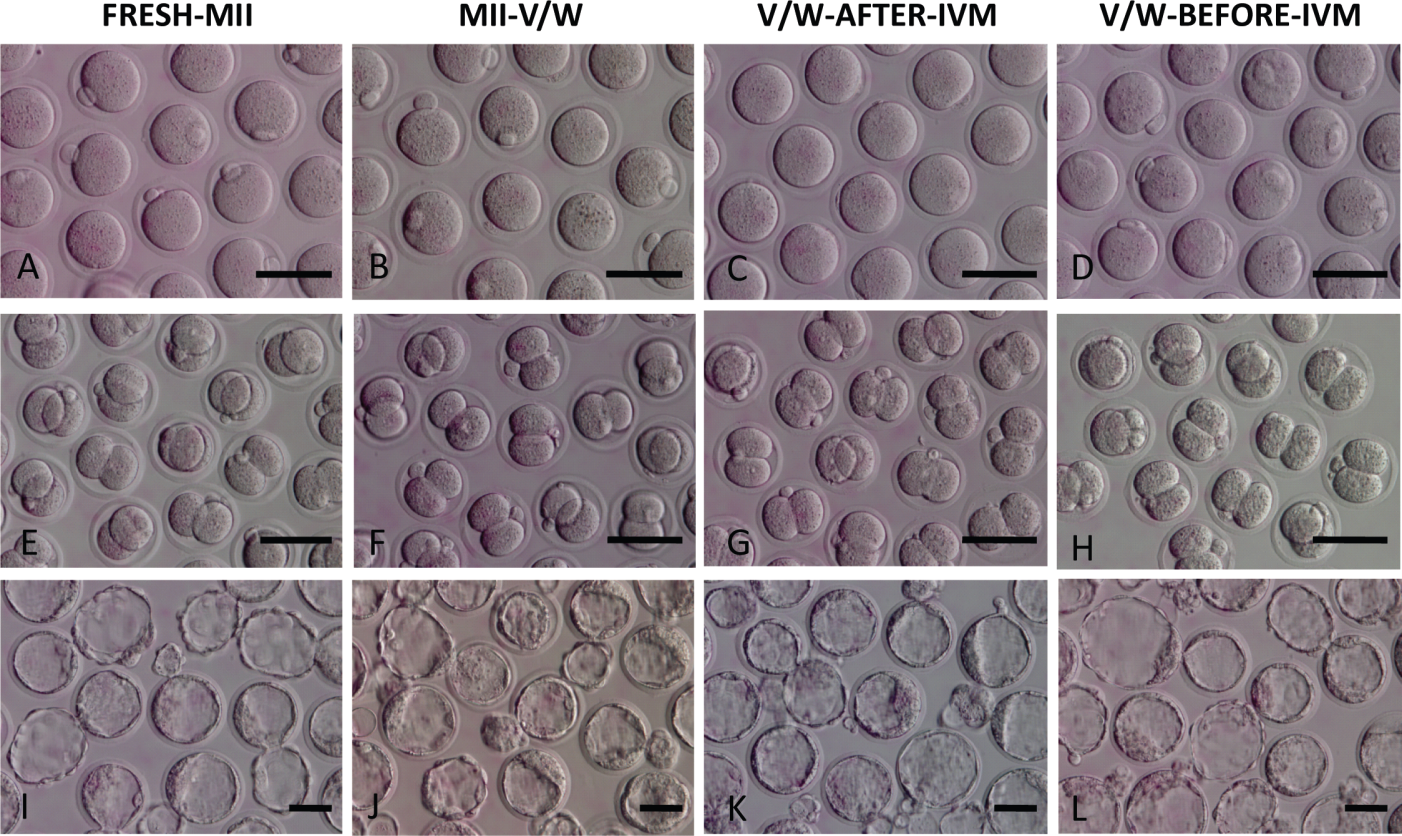
Preimplantation embryo development of FRESH-MII, VW-MII, V/W-AFTER-IVM and V/W-BEFORE-IVM oocytes following ICSI. (A) FRESH-MII oocytes. (B) MII-V/W oocytes after warming. (C) V/W-AFTER-IVM oocytes. (D) V/W-BEFORE-IVM oocytes. (E) Cleavage stage embryos derived from FRESH-MII oocytes. (F) Cleavage stage embryos derived from VW-MII oocytes. (G) Cleavage stage embryos derived from V/W-AFTER-IVM oocytes. (H) Cleavage stage embryos derived from V/W-BEFORE-IVM oocytes. (I) Blastocyst stage embryos derived from Fresh-MII oocytes. (J) Blastocyst stage embryos derived from VW-MII oocytes. (K) Blastocyst stage embryos derived from V/W-AFTER-IVM oocytes. (L) Blastocyst stage embryos derived from V/W-BEFORE-IVM oocytes. Scale bar = 100 μm.

We assigned oocytes in four treatment groups (Table 1). In FRESH-MII Group, a total of 517 IVO MII oocytes were collected and freshly subjected to ICSI. In MII-V/W Group, a total of 617 IVO MII oocytes were collected and vitrified. In V/W-AFTER-IVM Group, 790 MI stage oocytes were retrieved, out of which 664 matured (84.1%) after 8 h of *in vitro* culture. A total 589 out of 617 from MII-V/W Group (95.3%) and 644 out of 664 from V/W-AFTER-IVM Group (96.8%) MII oocytes survived after warming (MII-V/W Group vs. V/W-AFTER-IVM Group; NS; Table 1; Fig 3). In V/W-BEFORE-IVM Group, 818 MI oocytes were retrieved and vitrified, the MI oocyte survival rate was 92.4% (756/818) post vitrification/warming, and a total of 562 out of 818 starting MI oocytes were matured (68.7%) after 8 h of *in vitro* culture, significantly lower than that achieved in the V/W-AFTER-IVM Group (P<0.05). In vitrification groups, only viable and mature MII oocytes were selected for ICSI.

**Table 1:** *In vitro* development of vitrified oocytes after ICSI.

Group	No. of replicates	No. of starting MI oocytes	No. of MI oocytes survived post warming (% of per MI oocyte)	No. of available MII oocytes (% of per MI oocyte)	No. of oocytes survived post warming (% of per MII oocyte)	No. of available IVM MI-II oocytes post warming (% of per MI oocyte)	No. of oocytes survived post ICSI (% of per MII oocyte)	No. of cleaved embryos (% of per MII oocyte)	No. of blastocysts (% of per MII oocyte)
FRESH-MII (Control 1)	6	N/A	N/A	517	N/A	N/A	378 (73.3)^a^	376 (72.8)^a^	290 (56.1)^a^
MII-V/W (Control 2)	8	N/A	N/A	617	589 (95.3)^a^	N/A	344 (56.4)^b^	341 (55.8)^b^	274 (45.6)^b^
V/W-AFTER-IVM	9	790	N/A	664 (84.1)^a^	644 (96.8)^a^	644 (81.5) ^a^	339 (50.7)^b^	323 (48.3)^b^^c^	216 (32.4)^c^
V/W-BEFORE-IVM	8	818	756 (92.4)	562 (68.7)^b^	N/A	562 (68.7) ^b^	259 (45.9)^b^	252 (44.6)^c^	158 (27.5)^c^

Percentage data were arcsine transformed and subjected to Tukey's multiple comparisons test using Graphpad Prism (v6.02).

^a, b, c^ different superscripts within the same column indicates statistical difference (P<0.05).

To gain knowledge of the combinational effects of IVM and V/W on MI oocytes, we compared the ratio of number of viable MII oocytes over the starting number of MI oocytes. The overall efficiency was higher in V/W-AFTER-IVM than in V/W-BEFORE-IVM (81.5 vs. 68.7%; P<0.05; Table 1), suggesting that MII oocytes have better tolerance for vitrification than MI oocytes do.

MII oocytes in all groups were subjected to ICSI using sperm from B6D2F1 males. Survival rates post ICSI and embryo cleavage rates were checked after 24 h of ICSI. To allow comparison among these four groups, we used total available MII oocyte number as the denominator to calculate the rates (Table 1).

The survival rate post ICSI was highest in the FRESH-MII Group (P<0.05). No significant difference was found among the remaining three groups (Table 1; 56.4% vs. 50.7% vs. 45.9%; NS).

The order of subsequent embryo cleavage rates, from the highest to the lowest, FRESH-MII, MII-V/W, V/W-AFTER-IVM, and V/W-BEFORE-IVM (Fig 3; Table 1; 72.8% vs. 55.8% vs. 48.3%vs. 44.6%; p<0.05).

On Day 4 post ICSI, blastocyst formation was assessed (Fig 3, Table 1). The blastocyst formation rates were highest in FRESH-MII (56.1%), moderate in MII-V/W and V/W-AFTER-IVM (45.6% vs. 32.4%), and lowest in V/W-BEFORE-IVM (27.5%). As expected, the efficiencies of blastocyst formation in FRESH-MII Group was significantly higher than in the MII-V/W Group (56.1% vs. 45.6%, P<0.05; group FRESH-MII Group vs. MII-V/W Group), indicating V/W procedure adversely affected the capability of subsequent development. Further, the blastocyst rate was higher in MII-V/W (45.6%) than in V/W-AFTER-IVM (32.4%), confirming that in vivo matured oocytes possess higher developmental competency than *in vitro* matured ones.

Among *in vitro* matured groups, maturation prior to V/W (IVM-BEFORE-V/W) appeared to yield higher blastocyst rates than maturation post V/W (IVM-AFTER-V/W) (32.4% vs. 27.5%); however such difference is not statistically significant (P>0.05) when calculated based on the number of viable MII oocytes used for ICSI.

### The term development rates after embryo transfer

**Fig 4:**
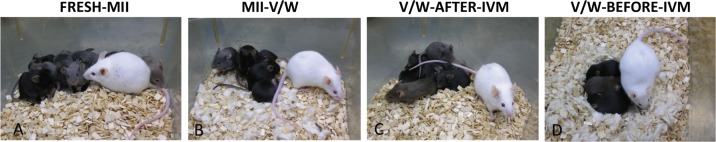
In vivo development of Fresh-MII, VW-MII, V/W-AFTER-IVM and V/W-BEFORE-IVM oocytes following ICSI. (A) 21 days-old offspring derived from Fresh-MII oocytes. (B) 17 days-old offspring derived from VW-MII oocytes. (C) 17 days-old offspring derived from V/W-AFTER-IVM oocytes. (D) 13 days-old offspring derived from V/W-BEFORE-IVM oocytes. Sources of both oocytes and sperms were derived from B6D2F1 strain; hence, the coat color of the ICSI pups (B6D2F2) showed various colors with gray, brown and black.

We next performed embryo transfer to evaluate the in vivo developmental competency of embryos derived using oocytes of all four groups (Table 2). A total of 80 blastocysts were transferred to 8 recipient mice in FRESH-MII Group, resulting in 7 pregnancies and 33 term pups. In MII-V/W Group, 90 blastocysts were transferred to 7 recipient mice, of which 5 became pregnant, delivering 21 term pups. In V/W-AFTER-IVM Group, 132 blastocysts were transferred to 9 recipient mice, of which 7 became pregnant and 22 pups were born. In V/W-BEFORE-IVM Group, 89 blastocysts were transferred to 6 recipient mice, of which 4 became pregnant and 11 pups were born (Fig 4).

**Table 2:** *In vivo* development of vitrified oocytes after ICSI.

Group	No. of recipient used	No. of pregnancy (%)	No. of embryos transferred	No. of offspring born(%)
FRESH-MII (Control 1)	8	7 (87.5)	80	33 (41.2)^a^
MII-V/W (Control 2)	7	5 (71.4)	90	21 (23.3)^b^
V/W-AFTER-IVM	9	7 (77.7)	132	22 (16.7)^b^
V/W-BEFORE-IVM	6	4 (66.7)	89	11 (12.4)^b^

Percentage data were arcsine transformed and subjected to Tukey's multiple comparisons test using Graphpad Prism (v6.02).

^a, b^ different superscripts within the same column indicates statistical difference (P<0.05).

We calculated the birth rates as the ratio of number of term pups over total embryos transferred. Birth rates were significantly higher in Fresh-MII Group (41.2%) than in the other three V/W groups (12.4–23.3%) (P<0.05), indicating that the influence of V/W is not limited till blastocyst stage but continue onto the in vivo embryo development.

Among the three V/W groups, the term rate was highest in MII-V/W Group (23.3%), followed by V/W-AFTER-IVM Group (16.7%), and V/W-BEFORE-IVM Group (12.4%), with no statistical difference among them (P>0.05).

Notably, satisfactory birth rates (12.4–16.7%) were achieved in both V/W-AFTER-IVM and V/W-BEFORE-IVM Groups. All offspring appear normal and healthy (Fig 4). These findings demonstrate the feasibility of using MI oocytes for production of live healthy offspring.

## Discussions

Oocyte vitrification is of basic and practical importance given the scarcity of female gametes in animals and in humans. We previously reported successful vitrification of MII and immature MI oocytes in mice, and derived embryonic stem cells (ntESCs) by nuclear transfer using the MII-V/W and V/W-BEFORE-IVM oocytes [12] but we didn’t examine their in vivo developmental capacities. To date, efficient vitrification of MII oocytes in a number of mammalian species including mice, rabbit, bovine, porcine, and humans has been achieved using a number of variants of vitrification protocols such as electro microscope grids [23], open pulled straw [24], Nylon/Cryo-loop [25], drops into liquid nitrogen, gel loading tips [26]. Vitrification of immature oocytes, however, remains to be a challenge. Development of an effective protocol to cryopreserve immature oocytes will significantly expand the available materials for fertility and developmental biology research, as well as for human IVF applications. In a routine cycle of controlled ovarian hyper-stimulation for ICSI, approximately 15–30% of retrieved oocytes are immature (i.e. MI & GV oocytes) [13, 14]. These immature oocytes are excluded from almost all clinical uses, representing a substantial waste of the female gametes.

The present work looked at the effects of vitrification on MI oocytes, both *in vitro* and in vivo. Satisfactory blastocyst rates and term rates were obtained using embryos derived from MI oocytes that were subjected to combinational treatments of IVM and V/W. To our knowledge, this is the first study reporting the generation of live offspring using V/W mouse IVM oocytes collected at MI stage. Our findings have several practical implications: (1) MI oocytes can be used for production of live healthy offspring, provided they are properly *in vitro* matured and V/W. (2) V/W can be performed before or after IVM. Together the present work demonstrates that by combining V/W and IVM, MI oocytes can be “rescued” for fertilization, resulting in satisfactory term development.

### The effects of companion cumulus cells

The blastocyst rate was lower in the V/W-AFTER-IVM group (32.4%) than in the MII-V/W group (45.6%) (Table 1), despite that the oocytes in both groups were V/W at MII stage. The compromised developmental competency of the V/W-AFTER-IVM group oocytes are likely caused by multiple factors. First, these oocytes were matured *in vitro* whereas the MII-V/W ones were in vivo matured. Second, perhaps more significantly, the oocytes in the V/W-AFTER-IVM group were denuded of cumulus cells upon retrieval (to determine their maturation status).

The oocyte communicates with and modifies its surroundings via direct physical contact with cumulus cells [27]. With the known roles of cumulus cells for optimal oocyte maturation, the lack of cumulus cells is one of many limitations in any IVM culture system using leftover oocytes from ICSI. The foundation of this relationship lies partly in highly specialized oocyte-cumulus cell contacts called trans-zonal projections (TZP) that are established at the onset of folliculogenesis [28]. It is known that removal of the cumulus oophorus before IVM can be detrimental to oocyte quality and subsequent embryo development [29–32]. Without the TZP between companion cumulus cells and oocytes, the denuded MI oocytes undergoing IVM are thus in a compromised micro-environment which is essential for complete oocyte maturation.

### The effects of oocyte stage at vitrification

A number of groups have reported effects of cryopreservation of GV stage oocytes. The effects of slow freezing are controversial: two studies showed that slow freezing at GV stage had better outcome than at post-IVM MII stage [16, 17], while several other studies reported low maturation rates when slow freezing at the GV stage was performed [18, 19, 33]. Vitrification at GV stage was reported to negatively affect maturation rates [34, 35].

The present work looked at effects of V/W and IVM on oocytes collected at the MI stage. It appears that V/W have similar impacts on the survival of MI oocytes (based on morphology observation), as compared to MII stage oocytes. As shown in Table 1, the oocyte survival rate was 95.3% for MII-V/W group, similar to that in the V/W-BEFORE-IVM group (92.4%) and that in the V/W-AFTER-IVM group (96.8%). Given that MI oocytes have similar surface to volume (S/V) ratio and cytoskeleton organization to those of MII oocytes, it is not surprising that when the same V/W protocols (i.e. same cooling rates and cryoprotectants) were applied, similarly high survival rates were obtained.

On the other hand, our data show that after V/W, the maturation rate dropped significantly (P<0.0001, Chi square test) to 74.3% (total matured/total survived after V/W, based V/W-BEFORE-IVM group data) vs. 84.1% in the V/W-AFTER-IVM group (Table 1), indicating that the V/W treatment negatively affected the IVM process. It remains to be tested what factors contributed to the difference. One possibility is the oocyte membrane permeability to the cryoprotectants may have changed along the maturation process, with MI oocytes having lower tolerance to cryoinjuries, consequently leading to comprised IVM outcome. Nevertheless, many healthy live offspring were produced in either group. Researchers can choose which protocol to use based on their preference and work context (e.g. available manpower, etc.).

## Conclusions

The present work demonstrates that MI oocytes are amenable to V/W. We show that combinational treatments of IVM and V/W on MI oocytes resulted in acceptable blastocyst development rates, and importantly, healthy offspring. These findings provide confidence and flexibility to researchers and clinical practitioners to store and use the otherwise discarded MI oocytes.
